# m7GRegpred: substrate prediction of N7-methylguanosine (m7G) writers and readers based on sequencing features

**DOI:** 10.3389/fgene.2024.1469011

**Published:** 2024-08-28

**Authors:** Yu Zheng, Haipeng Li, Shaofeng Lin

**Affiliations:** ^1^ Key Laboratory of Ministry of Education for Gastrointestinal Cancer, School of Basic Medical Sciences, Fujian Medical University, Fuzhou, Fujian, China; ^2^ School of Medical Technology and Engineering, Fujian Medical University, Fuzhou, China; ^3^ Graduate School of Fujian Medical University, Fuzhou, Fujian, China; ^4^ Department of Operating Room, Second Affiliated Hospital of Fujian Medical University, Quanzhou, China

**Keywords:** RNA modification, m7G, substrate, machine learning, webserver

## Abstract

N7-Methylguanosine (m7G) is important RNA modification at internal and the cap structure of five terminal end of message RNA. It is essential for RNA stability of RNA, the efficiency of translation, and various intracellular RNA processing pathways. Given the significance of the m7G modification, numerous studies have been conducted to predict m7G sites. To further elucidate the regulatory mechanisms surrounding m7G, we introduce a novel bioinformatics framework, m7GRegpred, designed to forecast the targets of the m7G methyltransferases METTL1 and WDR4, and m7G readers QKI5, QKI6, and QKI7 for the first time. We integrated different features to build predictors, with AUROC scores of 0.856, 0.857, 0.780, 0.776, 0.818 for METTL1, WDR4, QKI5, QKI6, and QKI7, respectively. In addition, the effect of window lengths and algorism were systemically evaluated in this work. The finial model was summarized in a user-friendly webserver: http://modinfor.com/m7GRegpred/. Our research indicates that the substrates of m7G regulators can be identified and may potentially advance the study of m7G regulators under unique conditions.

## 1 Introduction

Transcriptomics is a rapidly emerging frontier discipline in recent years, has made many advances. So far 170 RNA modifications have been identified (Boccaletto et al., 2022). N7-methylguanosine (m7G) is a widely present modified form of RNA molecules, mainly found in the 5 terminal end of cap structure and internal positions of mRNA in eukaryotes. In addition, it is also found in the interior of rRNA and tRNA of various species. Additionally, recent research has revealed that miRNAs are also subject to m7G methylation (Furuichi, 2015). Beyond its role in RNA’s standard physiological metabolic processes, m7G has played as a significant role in cancer research. Recent studies indicate that m7G and its associated regulatory proteins are considerably dysregulated in tumorigenesis (Katsara and Schneider, 2021). According to recent findings, the proportion of internal m7G/G in eukaryotic mRNAs typically falls between 0.02% and 0.05%, which is 5%–10% of the m6A/A ratio within mRNAs (Malbec et al., 2019).

As with various epigenetic modifications that have been examined, m7G on mRNA can bind with regulatory proteins, thus exerting pivotal roles in the complex biological processes of human cells. It is installed by the methyltransferases METTL1 and WDR4 (Vernet and Artzt, 1997). Additionally, three m7G readers, QKI5, QKI6, and QKI7, have been identified to preferentially recognize internal mRNA m7G modifications (Zhang et al., 2019), playing key roles in the regulation of RNAs carrying m7G.

The METTL1-WDR4 methyltransferase complex facilitates the formation of N (7)-methylguanosine on RNA with different category, including tRNA, mRNA, and microRNA (miRNA). Together, they mediate the m7G modification at the 46th position of tRNA. METTL1, the active unit of the complex, is a member of the Class I methyltransferase (MTase) family. Specifically, METTL1 is responsible for the addition of N (7)-methylguanosine at the 46th guanosine (m7G46) in a subset of tRNAs characterized by the presence of the 5′-RAGGU-3′ motif in the variable loop. This particular modification contributes to the stabilization of the tRNA’s tertiary structure and shields it from enzymatic degradation (Katsara and Schneider, 2021).

WDR4, on the other hand, is an inactive unit with the classic ring structure of the WD40 protein family, composed of seven repeated domains (B1-B7). METTL1 and WDR4 form a binary complex that provides a positively charged binding platform for the tRNA substrate. The tRNA is positioned at an angle on the complex, allowing its modification site (G46) to face the active pocket of METTL1. WDR4 serves as a scaffold, binding METTL1 and tRNA through its B3-B4 domains. Therefore, METTL1 requires the association with WDR4 to exert its normal methyltransferase activity (Li et al., 2023).

QKI belongs to the STAR domain family, a group of RNA-binding proteins (RBPs) with the K homology (KH) domain that plays a role in signaling pathways and RNA metabolism modulation (Zhao et al., 2023). The QKI gene encodes three major isoforms: QKI5, QKI6, and QKI7 (Darbelli and Richard, 2016). Research indicates that while QKI6 and QKI7 isoforms are mainly found in the cytoplasm, QKI5 is predominantly located in the nucleus. Stress granules (SGs) are non-membrane-bound cytoplasmic organelles formed under stress conditions, which can regulate mRNA stability, translation, storage, and may be linked to tumorigenesis and drug resistance. Recent studies have highlighted the significance of RNAs and their interacting RNA-binding proteins (RBPs) in stress granule formation. Under stress conditions, both QKI6 and QKI7 isoforms directly interact with G3BP1, a core component of stress granules leading to their co-localization within SGs. This interaction facilitates the sequestration of numerous internally m7G-modified mRNAs into stress granules impacting mRNA stability and translational efficiency (Zhao et al., 2023).

While numerous bioinformatics studies have collected RNA modification sites (Song et al., 2020; Ma et al., 2022; Zhang et al., 2023), and successfully predicted RNA modification sites in transcriptomics (Chen et al., 2019a; Chen et al., 2018; Chen et al., 2019b). For the prediction of m7G sites, about a dozen of predictor were developed and updated, by using sequence and structure features of m7G sites and training models with machine learning and deep learning (summarized in Supplementary Table S1). These state-of-the-art tools provide the accurate prediction of m7G sites, there has been a lack of focus on the specific substrates targeted by various m7G-related enzymes. Our study presents a new bioinformatics framework, “m7GRegpred,” which utilizes machine learning algorithms and sequence-based features to predict substrate specificity for m7G writers METTL1, WDR4, and readers QKI5, QKI6, and QKI7. Previous research has linked RNA modification and their regulators to various diseases (Song et al., 2023a; Wang et al., 2024; Song et al., 2023b). The m7GRegpred framework may aid in identifying substrates for each m7G regulator, offering insight into their roles in human diseases.

## 2 Methods and materials

### 2.1 The m7G sites and substrates of regulators

The m7GHub V2.0 database (Wang et al., 2024) provided data on m7G sites throughout the entire transcriptome, with 430,898 potential m7G sites identified in 23 species using both commonly used next-generation sequencing (NGS) and the newly developed Oxford Nanopore direct RNA sequencing (ONT) techniques. In this study, we utilized the 169,718 human m7G sites acquired from m7GHub V2.0 (Wang et al., 2024) and the binding regions of writers or readers from Gene Expression Omnibus (GEO) (Barrett et al., 2013) dataset (Table 1). The modification sites that overlapped with binding regions were identified as the substrates of m7G regulators.

**TABLE 1:** Sequencing results for identifying substrate of m7G regulators.

Regulators	Techniques	GEO accession	References
METTL1	PAR-CLIP	GSE100756	Bao et al. (2018)
		GSE112276	Zhang et al. (2019)
WDR4			
QKI5	RIP-seq	GSE193036	Zhao et al. (2023)
QKI6			
QKI7			

In our predictions, we identified positive sites as guanine sites within the substrate region of m7G regulatory factors, and maintained a 1:1 ratio by randomly selecting negative sites from unmethylated or unregulated guanine sites on the same transcript. Redundant sequences were removed using CD-HIT (Fu et al., 2012) software with default parameters. The non-redundant site data was then used to train the predictor, with 80% of the sites reserved for training and the remaining 20% for independent testing. Our prediction models were constructed using full transcript data, the unmodified and methylated sites from both exons and introns were considered.

### 2.2 Feature encoding methods

#### 2.2.1 Binary encoding method (OH)

Binary encoding method as known as one hot encoding is used to convert nucleotides in biological sequences into numerical form (Zhou et al., 2016). Each nucleotide (A, C, G, U) is encoded as a four-bit binary vector. For example, the sequence UACCGU are converted into binary vectors {(0,0,0,1), (1,0,0,0), (0,1,0,0), (0,1,0,0), (0,0,1,0), (0,0,0,1)}.

#### 2.2.2 Nucleic acid composition (NAC)

Within the scope of our research, we have harnessed the frequencies of dinucleotide pairs for the encoding of sequences, encapsulated in a 16-dimensional feature vector encompassing combinations from AA to UU. The feature vector $F_{i}$ is defined as $\left(f_{AA},f_{Ac},f_{AG},\ldots \ldots f_{UU},\right)$ . Wherein the symbol f signifies the relative incidence of the dinucleotide within the *i*-th sequence.

#### 2.2.3 Accumulated nucleotide frequency (ANF)

This method breaks down each sequence into individual nucleotides and generates a feature at each position within the sequence. Each feature represents the cumulative occurrence of the nucleotides at that position in the sequence. The *i*-th nucleotide feature is determined by the frequency of that nucleotide appearing in the initial *i* nucleotides of the sequence.

#### 2.2.4 Pseudo k-tuple composition (PKC)

In the bioinformatic field, PKC has gained widespread adoption as an encoding method, encompassing applications in protein, DNA, and RNA prediction (Feng C. Q. et al., 2019; Feng P. et al., 2019; Guo et al., 2014; Liu et al., 2018a; Liu et al., 2018b; Su et al., 2018). Numerous bioinformatics tools, web-based platforms, and software packages have incorporated PKC methods into their repertoire. In this study, we applied the PKC method to encode RNA sequence from online servers (Chen Z. et al., 2021).

#### 2.2.5 Chemical property (CP)

The encoding of nucleotide sequences is achieved by leveraging three distinct chemical properties: the presence of ring structures, the type of functional groups, and the capacity for hydrogen bonding. Specifically, adenine (A) and cytosine (C) are characterized by an amino group, in contrast to guanine (G) and uracil (U), which feature a keto group. A and G possess a double-ring structure, while C and U are distinguished by a single ring. During the process of hybridization, A and U are capable of establishing two hydrogen bonds, but triple hydrogen bond happens in G and C hybridization. Utilizing these chemical attributes, A = (1,1,1), C = (0,1,0), G = (1,0,0), U = (0,0,1).


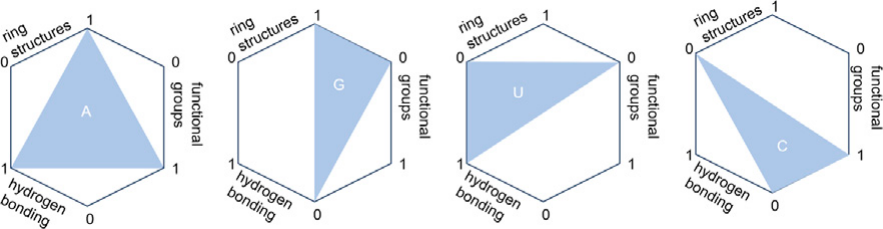


#### 2.2.6 Electron-ion interaction pseudopotential (EIIP)

Different Nucleic acids have different electron-ion interaction potential values, which follows: A is 0.1260, U is 0.1335, C is 0.1340, and G is 0.0806. This method can transform each nucleic acid in an RNA sequence into a numeric vector corresponding to the EIIP values. For example, “AUCG” will be converted into (0.1260, 0.1335, 0.1340, 0.0806).

### 2.3 Algorisms and evaluation

Machine learning algorithms have powerful data processing and pattern recognition capabilities, making them widely used in the field of biological research, particularly in DNA, RNA and protein modification prediction. In this study, we trained all models with R programme (Version 4.3.3) and corresponding packages for each m7G regulator, and use Support Vector Machine (SVM, e1071 package (Meyer et al., 2020)) to construct predictive models that are tuned to form the final predictor. For the feature selection of six encoding methods, all 63 combinations of single or multiple feature encoding methods were individually used to train and test, and the feature combination with best performance was selected for further improvement. In addition to the feature encoding, the sequence length is important for predictor. For the selection of sequence length, we tested 21, 41, 61, and 81 nucleotides (nt), centered on the m7G modification site.

In order to determine the most appropriate algorithm to build the model, we systematically compared other three of the more popular machine learning algorithms, including Random Forest (RF, randomForest (Liaw and Wiener, 2001)), Generalized Linear Model (GLM, stats package (R Core Team, 2011)), and efficient extreme gradient boosting (XGBoost, xgboost package (Chen and Guestrin, 2016)). We assessed the performance of the predictor through independent tests, in addition to testing the effect of parameter selection. The main metric used to assess performance was AUROC (Area Under the Receiver Operating Characteristic Curve). We also calculated Accuracy (ACC), Sensitivity (Sn) and Specificity (Sp) to compare algorithm performance.

### 2.4 The design and construction of m7GRegpred website

The framework of m7GRegpred website was structured into two primary components, including the core module for prediction and adjuvant interface. For the former, the “Prediction,” “Controller” and “Results” collectively constitute the prediction system of m7GRegpred. For the latter, the “Home,” “Guide,” “Download” and “Contact” are major functional page for providing the associated information of m7GRegpred. The pages of m7GRegpred webserver were developed through the amalgamation of HTML5, CSS, JavaScript, PHP, and JavaScript library (jQuery), and the “Results” page used the DataTables, a JavaScript library, to provide interactive visualization of predicted results.

## 3 Results

### 3.1 Data statistics and function analysis of m7G regulator-specific substrates

**FIGURE 1:**
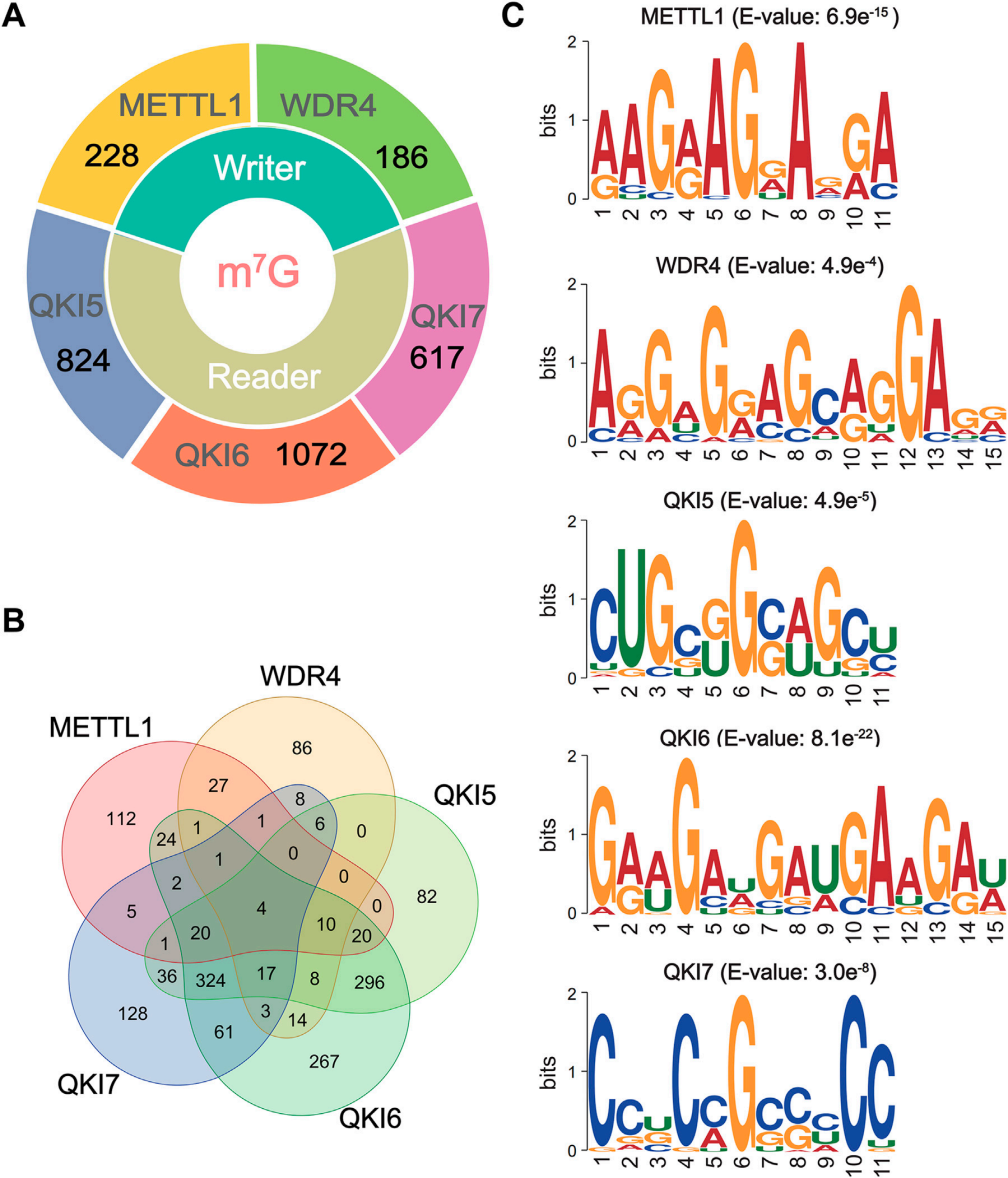
The data statistics and motif analysis of m7G regulator-specific substrates. **(A)** The number of known substrates of m7G regulators. The details are shown in Supplementary Table S2. **(B)** The Venn diagram of m7G substrates for m7G regulators by R package, ggvenn. **(C)** The major motifs of substrates for m7G regulators using XSTREME.

Recent research results have indicated the reliability and efficacy of sequence-derived features in reflecting the intrinsic specificity of target sequences. Therefore, we conducted an exploration of six different encoding methods to compare the efficacy of different encoding methods in predicting the substrate specificity of the m7G regulators. After de-redundancy by CD-HIT (Fu et al., 2012) filter, within the full transcriptome model, a total of 228, 186, 824, 1,072, and 617 sequences were considered as substrates for METTL1, WDR4, QKI5, QKI6, and QKI7, respectively (Figure 1A; Supplementary Table S2). Then, the Venn diagram of substrates across five m7G regulator indicated a low coverage between two m7G writers or among three m7G readers (Figure 1B). Only 44 sites were modified by both METTL1 and WDR4, and 365 sites were recognized by 3 m7G readers (Figure 1B). Furthermore, the motif analysis of m7G regulator-specific substrates was performed by using XSTREME (Bailey, 2021) with default parameters. The m7G writers exhibit diverse patterns, the substrates of METTL1 showed a “GxAG” motif, while WDR4 recognize the “GxxxGA” motif (Figure 1C). For m7G readers, QKI5 and QKI6 showed a similar motif of “GxxG,” whereas QKI7 recognize the “CxG” motif (Figure 1C). The results indicated that different m7G regulator recognized different sequence preferences for substrate m7G (Figures 1B,C).

**FIGURE 2:**
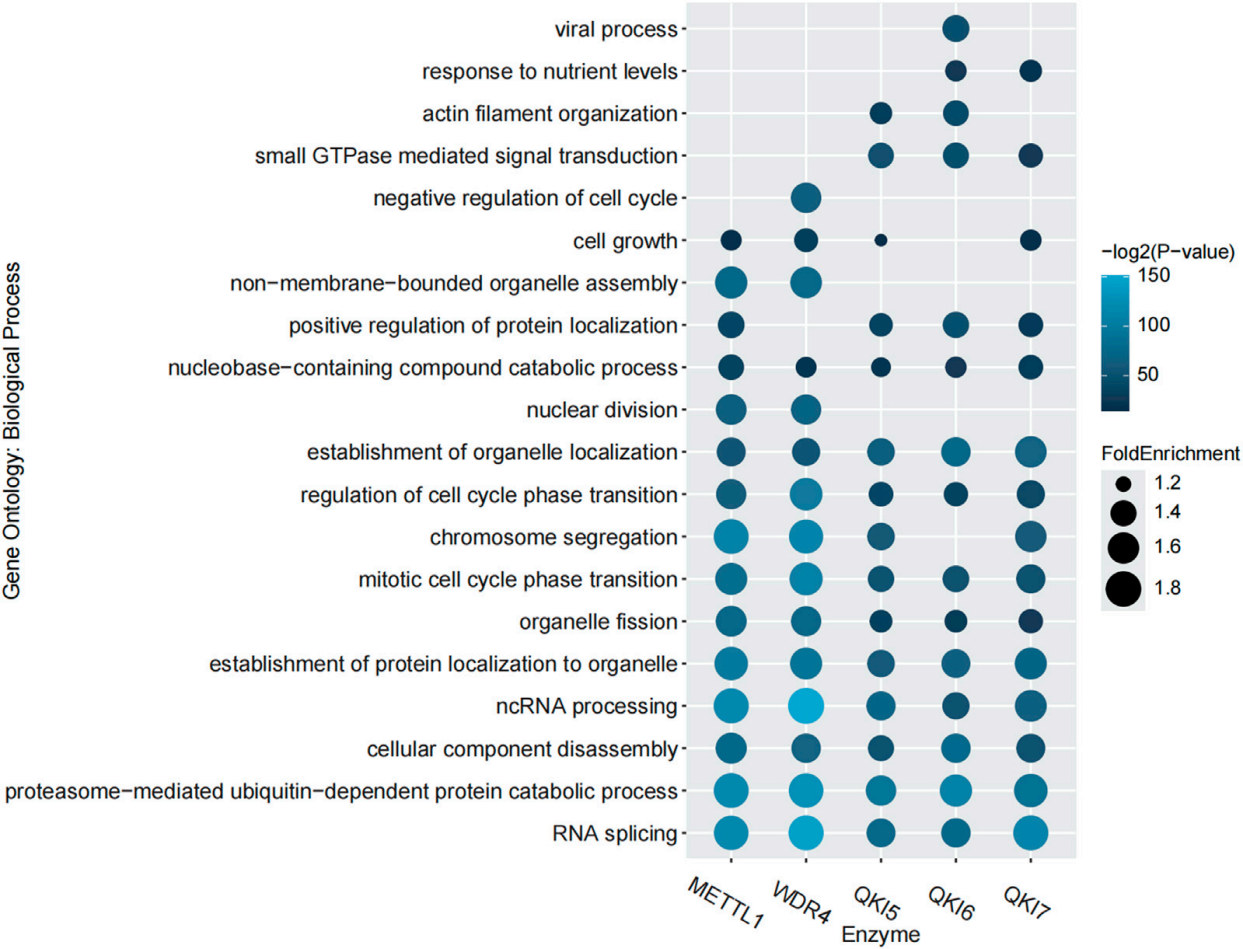
GO enrichment analysis of the regulator substrates. The top 15 GO terms of each regulator are shown.

In order to explore the correlation between m7G regulator substrates, m7G modifications and biological functions, we performed Gene Ontology (GO) enrichment analysis using the R package, ClusterProfiler (Wu et al., 2021). With this approach, we were able to identify biological processes associated with m7G regulators and reveal their potential roles in cellular functions. Figure 2 displays the top fifteen relevant GO biological process terms corresponding to each regulator substrate. The substrates of METTL1 and QKIs in particular showing significant enrichment under the GO term “positive regulation of protein localization,” which may indicate their key roles in regulating protein transport and localization.

### 3.2 Features and performances

**FIGURE 3:**
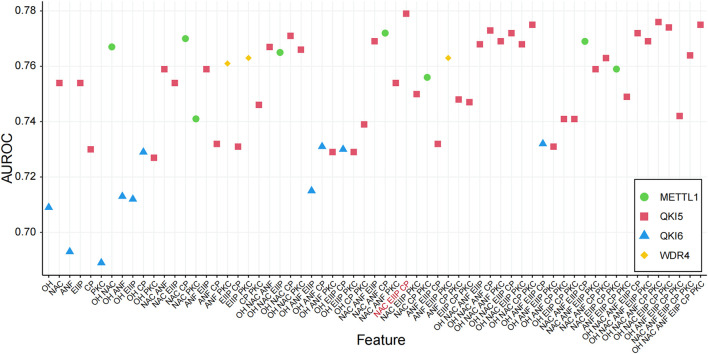
Comparison of different combinations of features. AUROC values of all feature combinations on independent dataset were evaluated (Supplementary Table S3), and selected the feature combination with the best prediction: CP: Chemical property, EIIP: Electron-ion interaction pseudopotential, NAC: Nucleic acid composition.

We performed the model training on the training data for each m7G regulator, followed by validation on the independent test data, to determine the best features predicted by the m7G regulators by calculating the AUROC values (Table 1). We considered all possible combinations of features across six encoding schemes, ranging from single features to multiple feature combinations for each regulator (Supplementary Figure S1; Supplementary Table S3). The results indicated that the feature combinations from the NAC, EIIP, and CP encoding methods exhibited the best performance in the prediction of regulators and substrates (Figure 3). We demonstrate the performance (AUROC) of various feature combinations in the prediction of substrate regulation across several substrates. Consequently, we selected the NAC, EIIP, and CP encoding methods to construct the preliminary m7GRegpred, the three-feature combination and selection framework demonstrates higher accuracy, surpassing the performance of any individual sequence-derived feature (Table 2; Supplementary Table S3).

**TABLE 2:** The AUROC values on independent test with individual features and selected feature combination. All AUROC values of independent test for different combinations of features is shown in Supplementary Table S3.

Feature	METTL1	WDR4	QKI5	QKI6	QKI7
OH	0.669	0.597	0.691	0.709	0.660
NAC	0.737	0.735	0.754	0.696	0.697
ANF	0.639	0.602	0.666	0.693	0.637
EIIP	0.737	0.735	0.754	0.696	0.697
CP	0.684	0.629	0.730	0.721	0.683
PKC	0.669	0.687	0.659	0.689	0.672
NAC, EIIP, and CP	0.774	0.769	0.779	0.732	0.718

### 3.3 Performance of different length windows

**FIGURE 4:**
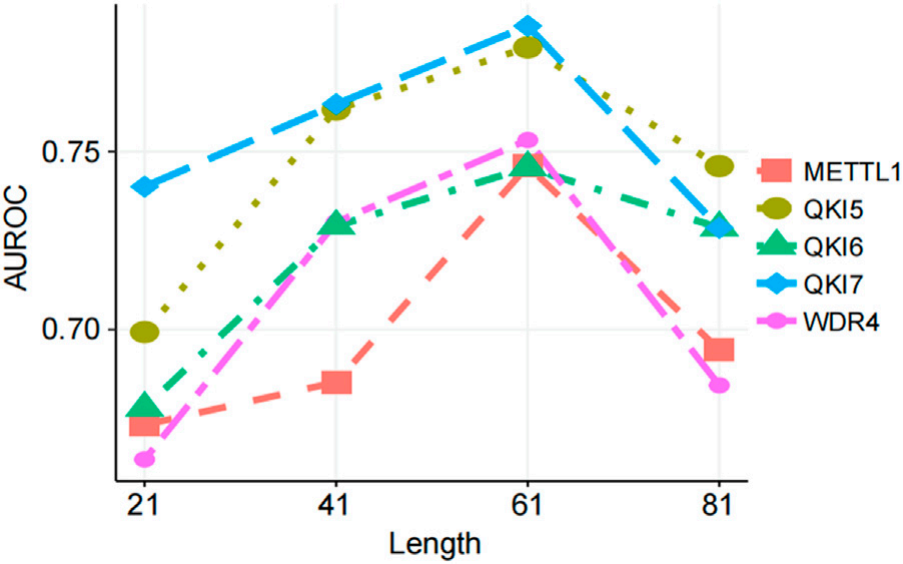
Prediction performance with different window lengths. Performance of CP, EIIP, and NAC feature combination encoding method comparing windows of different lengths.

Different lengths of sequence window lengths contain different amounts of sequence information, and the choice of length directly affects the performance of the trained predictor (Chen K. et al., 2021; Chen et al., 2015). Therefore, we chose reasonable length of the input sequence after comprehensive consideration. We tested sequences centered on the m7G modification site with lengths of 21, 41, 61, and 81 nucleotides (nt) to determine the optimal predictive results (Figure 4). In the full transcriptome model, the substrate prediction performance for several regulators initially improved. As the length increased, the AUROC score reached its highest value, and then the AUROC score gradually decreased or leveled off. Based on these results, we selected a sequence length of 61 nt in the full transcriptome model for several regulators to generate features (Figure 4).

### 3.4 Algorisms and evaluation

**FIGURE 5:**
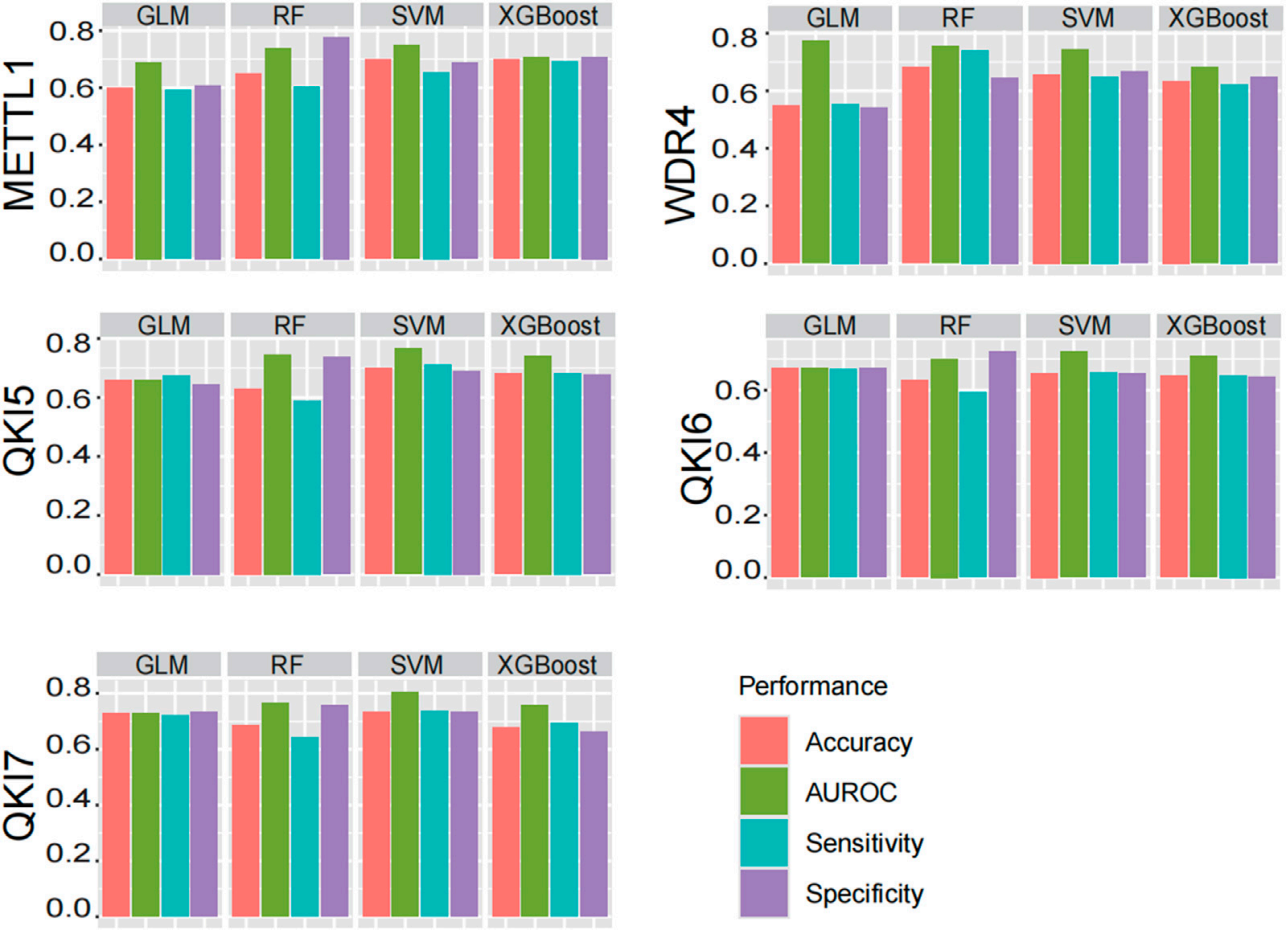
Performance comparison of different machine learning algorithms. Four commonly used machine learning algorithms are compared: SVM, RF, GLM, XGBoost. And AUROC, Acc, Sn, Sp are evaluated, and finally SVM is chosen as the model.

Support Vector Machine (SVM) have been widely recognized and applied in the field of RNA modification prediction due to their excellent prediction accuracy and generalisation ability (Chen et al., 2019a; Chen et al., 2019b; Chen K. et al., 2021; Chen et al., 2015; Liu et al., 2020; Huang et al., 2018; Chen et al., 2016; Feng et al., 2017). In order to confirmed that SVM is a more suitable machine learning algorithm for our project to perform prediction of substrate of regulators, we conducted a systematically comparison with other prominent algorithms, including RF, GLM, and XGBoost (Figure 5). The performance of the predictors was assessed primarily by calculating AUROC on the independent test data, and also by evaluating metrics such as Accuracy, Sensitivity and Specificity to aid judgement of the performance of the predictors. In sum, when employing an optimized sequence length, SVM demonstrated the most consistent and superior performance across the board.

### 3.5 Parameter optimization of the SVM model

The parameter settings of the SVM model will affect the model’s predictive performance. The parameters we commonly adjust for SVM are Cost (C) and Gamma. The C parameter is a key hyperparameter that controls the model’s tolerance to misclassification. Setting a higher value of C increases the model’s fit to the training data, but this may also cause overfitting and make the model less able to generalise. Meanwhile, the gamma parameter determines the coverage of the Radial Basis Function (RBF) kernel, affecting the model’s sensitivity to local variations. Therefore, reasonable tuning of the C and gamma parameters is essential to achieve optimal performance of the SVM model.

**FIGURE 6:**
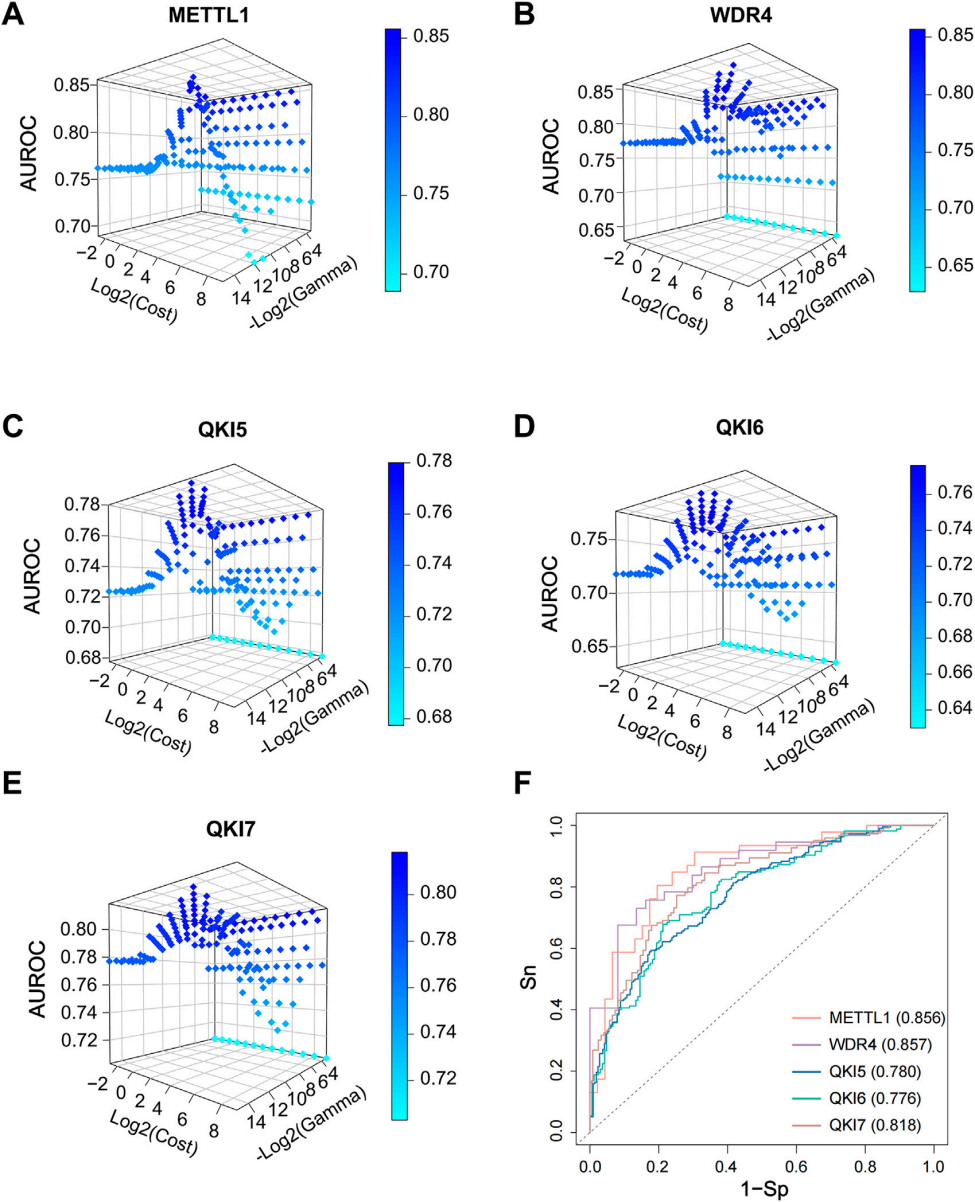
SVM model tuning parameters on an independent set. **(A–E)** Adjust the parameters of the SVM model, with the *x*-axis representing the logarithm base two of the cost values (log2 (cost)), and the y-axis representing the negative logarithm base two of the gamma values (-log2 (gamma)), with the y-axis also indicating the corresponding AUROC values. **(F)** The ROC curves and AUROC values of independent dataset for final model.

In this study, we combined all the parameter combinations for the C parameter range from 2 ^ (−3) to 2 ^ 9 and the Gamma parameter range from 2 ^ (−15) to 2 ^ (−3) and used this to compare the predictive performance of the models by calculating the AUROC values (Figures 6A–E). Based on the resulting AUROC values, the most appropriate parameter combinations were selected. Specifically, the final optimized model achieved AUROC scores of 0.856, 0.857, 0.780, 0.776, and 0.818 in the independent tests for substrate prediction of the full transcriptome model for METTL1, WDR4, QKI5, QKI6, and QKI7, respectively (Figure 6F).

### 3.6 Model cross-validation prediction

**FIGURE 7:**
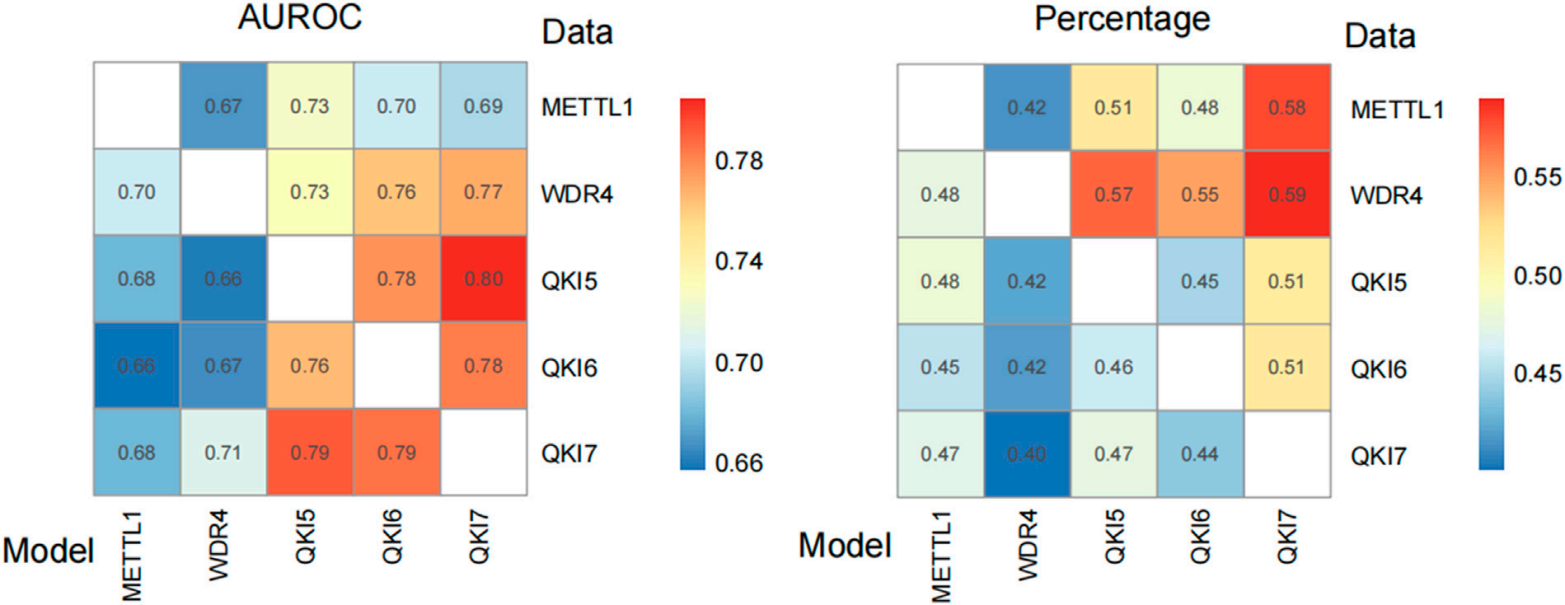
Cross-prediction of predictive models and m7G regulator substrate data. The values represent the predictive efficacy in terms of AUROC values (Left). The values indicate the ratio of sites predicted to be positive to all regulator substrate site (Right). The values were visualized by R package, pheatmap.

Based on the optimized predictors obtained above, the cross-talk between among five regulators were estimated (Figure 7). The high AUROC scores suggest most m7G sites could be regulated by the METTL1/WDR4 and recognized by the proteins from QKIs family. Please also notice that, the higher performance of QKIs or METTL1/WDR4 model predict substrate of themselves than METTL1/WDR4 predict QKIs suggest the there are some new writers and readers participated the regulation of m7G, which should be identified in further.

### 3.7 Webserver of m7GRegpred

**FIGURE 8:**
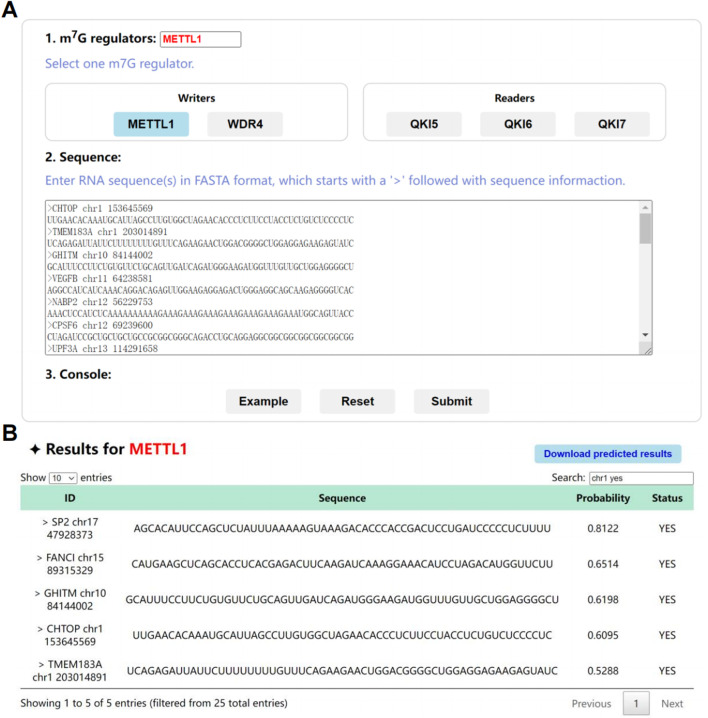
The webserver of the m7Gregpred. **(A)** The interface of “Prediction” page with the example, by clicking the “Example” button. **(B)** The filtered results of the prediction of selected m7G regulator and submitted sequences.

The web server of m7GRegpred was developed with a user-friendly manner (Figure 8), and structured into the kernel prediction component and adjuvant interfaces. The concise and interactive pages of online server used the amalgamation of HTML5, CSS, PHP, JavaScript, and JavaScript libraries (jQuery and DataTables). Users can select an interesting m7G writer or reader, and upload single or multiple RNA sequences (61 nt) in FASTA format. After the clicking of “Example” button of console, the m7G writer METTL1 and multiple RNA sequences will be loaded as an example (Figure 8A). After submitting, the predicted results of selected m7G regulator will be visualized, the probabilities of submitted sequences are shown in an interactive table (Figure 8B). Also, the predicted results can be directly downloaded as a text file, to further process for users without login requirement (Figure 8B).

## 4 Conclusion

In this study, we present a full-transcriptome prediction model based on the SVM algorithm designed to identify potential substrates for m7G regulatory factors. The model covers a series of key m7G regulators, including METTL1, WDR4, QKI5, QKI6, and QKI7. We selected six sequence encoding methods in pursuit of the most effective feature combination and ultimately chose features constituted by a combination of three sequence encoding methods to train the model. Our framework achieved high performance on independent test sets. Subsequently, we constructed predictors using different subsets of sequence-derived features for comparison. The results showed that this framework has higher performance than traditional sequence encoding methods. After comparing different machine learning algorithms, we ultimately chose SVM as the model. We then adjusted the parameters of the SVM model, compared the predictive effects of different parameter combinations, and selected the optimal predictive parameters to construct the final predictive model. Finally, GO enrichment analysis was performed to explore the similar biological functions of substrate of METTL1, WDR4 and QKI5, QKI6 and QKI7 from the QKI family, which suggest our processed training data is appropriate and consistent with known biological knowledge.

We also provide a website for proper sharing of our prediction models, which can be used by users for m7G regulator substrate prediction. This tool enables researchers to pinpoint m7G regulators sites in the transcriptome, providing a means to understand the regulatory mechanisms and biological functions affected by m7G regulators. May potentially advance the study of m7G regulators under unique conditions.

Despite the progress made in this study in predicting substrates of m7G regulators, we recognise that there are some limitations. Firstly, although predictors based on sequence-derived features have demonstrated an acceptable level of performance, in order to further improve prediction accuracy and reliability, future research should consider more advanced genomic features in combination (Chen K. et al., 2019). Recent studies have indicated that deep learning algorithms are effective in site prediction (Song et al., 2021; Huang et al., 2022). Therefore, incorporating additional genomic features or applying deep learning algorithms may help to improve performance.

## Data Availability

Publicly available datasets were analyzed in this study. This data can be found here: http://modinfor.com/m7GRegpred/.
