# Supplementary figures and images for: m7GRegpred: substrate prediction of N7-methylguanosine (m7G) writers and readers based on sequencing features
Source: Front Genet. 2024 Aug 28;15:1469011. doi: 10.3389/fgene.2024.1469011 (PMC11387174; doi:10.3389/fgene.2024.1469011)

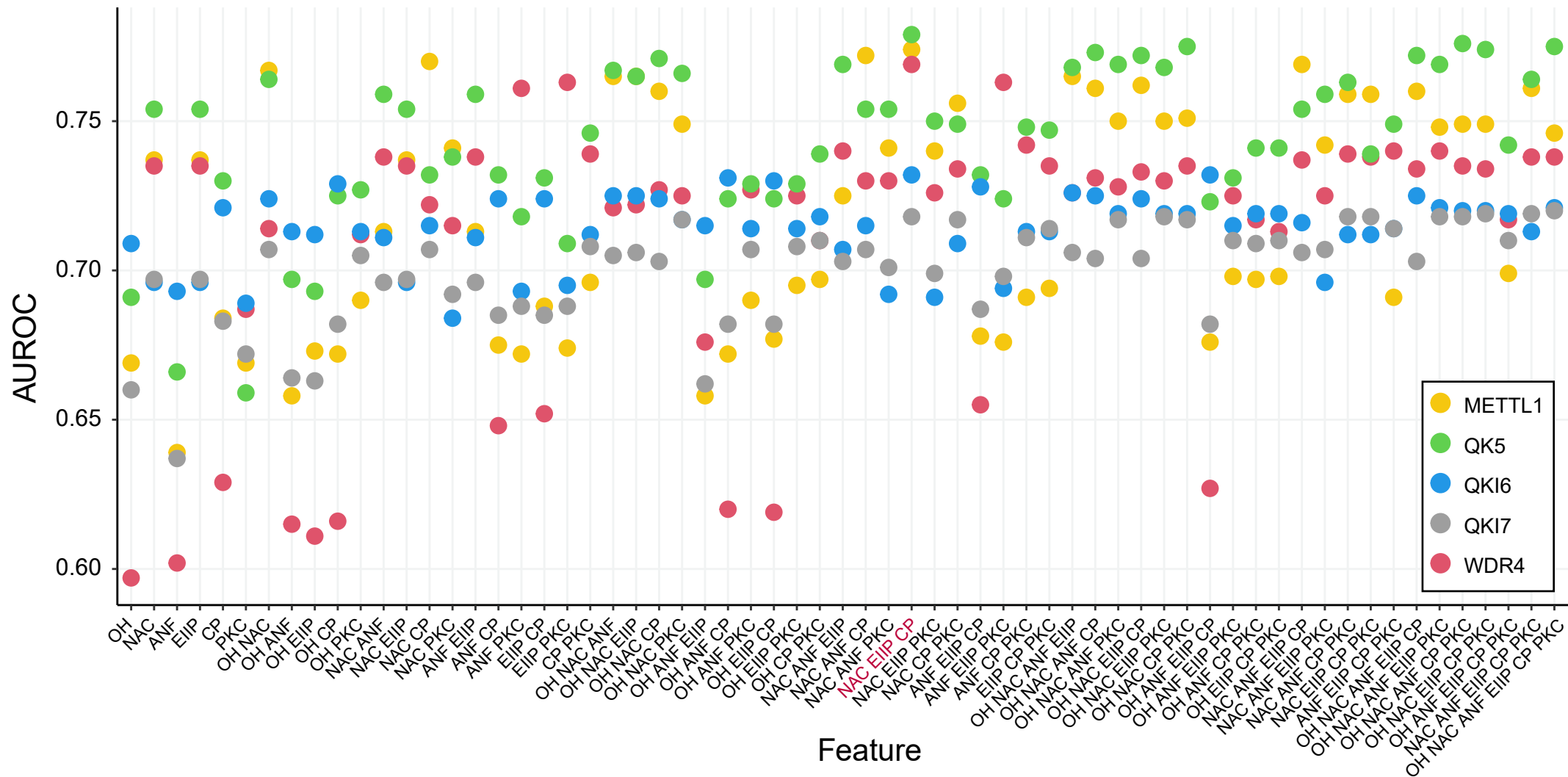

Supplement: Supplementary file 2 [file Image1.PDF]
